# The Role of Lipids in Parkinson’s Disease

**DOI:** 10.3390/cells8010027

**Published:** 2019-01-07

**Authors:** Helena Xicoy, Bé Wieringa, Gerard J. M. Martens

**Affiliations:** 1 Department of Cell Biology, Radboud Institute for Molecular Life Sciences (RIMLS), Radboudumc, 6525 GA Nijmegen, The Netherlands; Helena.xicoy@radboudumc.nl (H.X.); Be.Wieringa@radboudumc.nl (B.W.); 2 Department of Molecular Animal Physiology, Donders Institute for Brain, Cognition and Behaviour, Radboud University, 6525 GA Nijmegen, The Netherlands

**Keywords:** Parkinson’s disease, fatty acyls, glycerolipids, glycerophospholipids, sphingolipids, sterol lipids, lipoproteins, α-synuclein-mediated pathology, disease-modifying effects, neuroprotection

## Abstract

Parkinson’s disease (PD) is a neurodegenerative disease characterized by a progressive loss of dopaminergic neurons from the nigrostriatal pathway, formation of Lewy bodies, and microgliosis. During the past decades multiple cellular pathways have been associated with PD pathology (i.e., oxidative stress, endosomal-lysosomal dysfunction, endoplasmic reticulum stress, and immune response), yet disease-modifying treatments are not available. We have recently used genetic data from familial and sporadic cases in an unbiased approach to build a molecular landscape for PD, revealing lipids as central players in this disease. Here we extensively review the current knowledge concerning the involvement of various subclasses of fatty acyls, glycerolipids, glycerophospholipids, sphingolipids, sterols, and lipoproteins in PD pathogenesis. Our review corroborates a central role for most lipid classes, but the available information is fragmented, not always reproducible, and sometimes differs by sex, age or PD etiology of the patients. This hinders drawing firm conclusions about causal or associative effects of dietary lipids or defects in specific steps of lipid metabolism in PD. Future technological advances in lipidomics and additional systematic studies on lipid species from PD patient material may improve this situation and lead to a better appreciation of the significance of lipids for this devastating disease.

## 1. Introduction

Parkinson’s disease (PD) is the second most common neurodegenerative disease affecting 1% of the population above 60 years and up to 4% of individuals in the highest age groups [1]. Parkinson’s disease is characterized by motor symptoms, such us tremor, rigidity, bradykinesia (slowed movement) and impaired balance [2], and non-motor manifestations, including sleep disorders, and autonomic, gastrointestinal, sensory, and neuropsychiatric symptoms [3]. These symptoms are associated with a progressive loss of dopaminergic (DA) neurons from the nigrostriatal pathway, formation of Lewy bodies (LB), and microgliosis [4]. In familial PD, which explains 5–10% of all cases, these abnormalities may be caused by a mutation in one of the thus far known 19 familial genes, including *SNCA*, *LRRK2*, *PRKN*, *PINK1* and *DJ-1*, among others [5]. The remaining 90–95% of PD cases are of sporadic nature with unknown etiology.

Despite a large number of studies on familial forms of PD or toxin-induced cell and animal PD models (e.g., use of 1-methyl-4-phenyl-1,2,3,6-tetrahydropyridine (MPTP), rotenone or 6-hydroxydopamine (6-OHDA)) [6,7,8], no disease-modifying treatment for PD has been developed yet. Thus, additional approaches are necessary to advance the field of PD. Previously, we used data from genome-wide association studies and other genetic studies of PD patients to build a molecular landscape [9]. This enabled us to identify, in an unbiased way, various processes and pathways that might be involved in PD. Interestingly, we found that lipids play a key role in most of the processes that have been (classically) associated with PD (i.e., oxidative stress, endosomal-lysosomal function, endoplasmic reticulum stress, and immune response), and thus in PD etiology. In agreement with this observation, not only mutations in the gene encoding the lipid-producing enzyme glucocerebroside (*GBA*) are associated with familial PD [10,11,12], but also multiple single-nucleotide polymorphisms (SNPs) located in other genes involved in lipid metabolism, e.g., *SREBF1* [13], *DGKQ* [14], *ASAH1* [15] or *SMPD1* [16], have been linked to sporadic PD.

**Figure 1:**
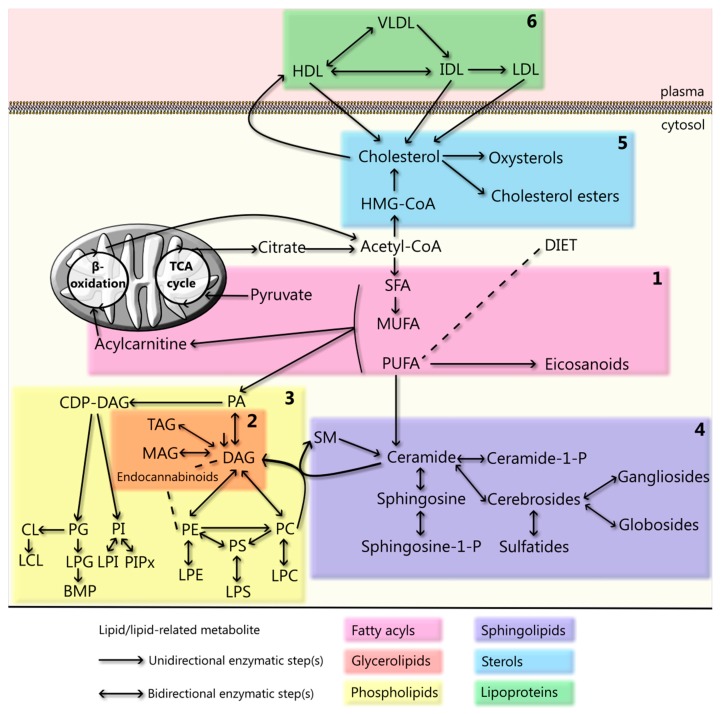
Cellular lipid metabolism and lipoprotein cycle. Schematic representation of lipid metabolism, whereby each colored box represents one lipid class: (**1**) fatty acyls, which include saturated (SFA), monounsaturated (MUFA), and polyunsaturated (PUFA) fatty acids, their mitochondrial-transporter, acylcarnitine, and the PUFA-derivatives eicosanoids; (**2**) glycyerolipids, including monoacylglycerol (MAG), diacylglycerol (DAG), and triacylglycerol (TAG), together with endocannabinoids (even though only some of them belong to this lipid class); (**3**) phospholipids, which include phosphatidic acid (PA), phosphatidylcholine (PC), phosphatidylserine (PS), phosphatidylethanolamine (PE), phosphatidylinositol (PI), phosphatidylglycerol (PG), cardiolipin (CL), and their lyso derivatives (lysoPC (LPC), lysoPS (LPS), lysoPE (LPE), lysoPI (LPI), lysoPG (LPG) and lysoCL (LCL)), and Bis(monoacylglycero)phosphate (BMP); (**4**) sphingolipids, including ceramide(-1-phosphate), sphingosine(-1-phosphate), sphingomyelin (SM), cerebrosides, sulfatides, gangliosides, and globosides; (**5**) sterols, which include the metabolites of cholesterol synthesis, such as β-hydroxy β-methylglutaryl-CoA (HMG-CoA), cholesterol, and its derivatives cholesterol esters and oxysterols; and (**6**) lipoproteins, including high-density lipoproteins (HDL), intermediate-density lipoproteins (IDL), low-density lipoproteins (LDL), and very low-density lipoproteins (VLDL). A depiction of the various lipid structures and of all the metabolic steps involved in their generation and interconversion(s) is given in Figures 2a,b–6a,b, respectively.

Lipids are biomolecules soluble in nonpolar organic solvents, usually insoluble in water, and primarily known for their metabolic role in energy storage [17,18]. Furthermore, they are the main constituents of cellular membranes, part of membrane rafts and protein anchors, and signaling and transport molecules [19,20,21,22,23]. There are eight different classes of lipids, classified as fatty acyls, glycerolipids, glycerophospholipids, sphingolipids, sterols, prenols, saccharolipids, and polyketides [24]. Here we will review the current knowledge of the role of the first five lipid classes and of lipoproteins in PD (Figure 1). Certain aspects of the relationship between PD and lipids are beyond the scope of this review, including the complex interaction between (membrane) glycerophospholipids and α-synuclein, the interaction between lipid classes, and the role of cholesterol derivatives, such as bile acids, tocopherols, and tocotrienols (vitamin E), vitamin A and carotenoids, vitamin D, steroidal hormones (e.g., estrogen) and coenzyme Q10.

## 2. Fatty Acyls

**Figure 2:**
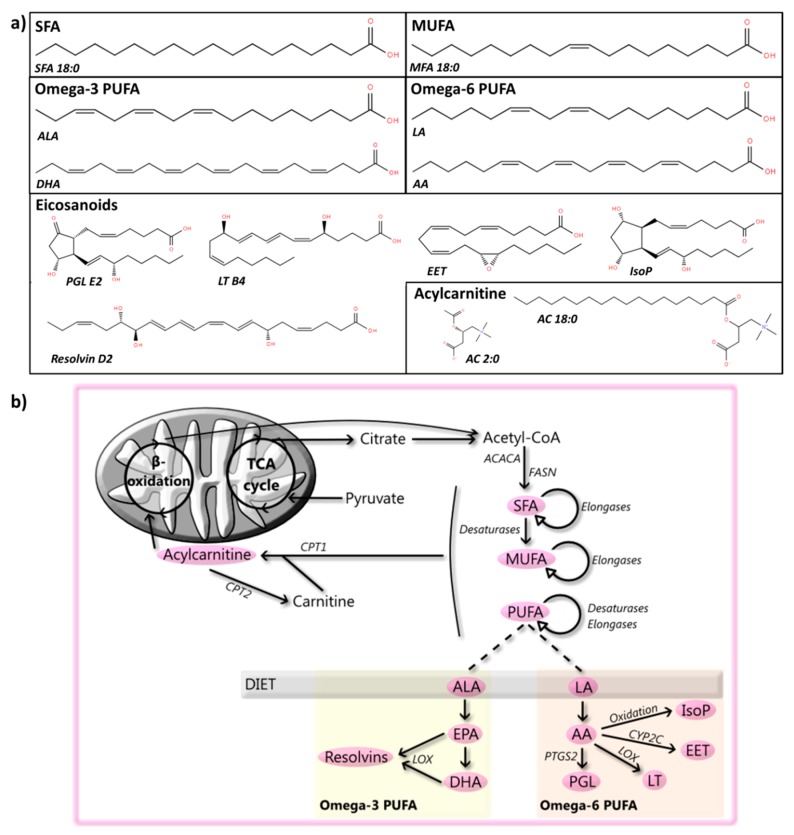
Fatty acyls: structures and metabolic steps involved. (**a**) Schematic representation of the chemical structures of fatty acyls, including saturated fatty acids (SFA 18:0), monounsaturated fatty acids (MUFA 18:1), omega-3 polyunsaturated fatty acids (PUFA, alpha-linoleic acid (ALA, top) and docosahexaenoic acid (DHA, bottom)), omega-6 PUFA (linoleic acid (LA, top) and arachidonic acid (AA, bottom)), eicosanoids (from left to right, prostaglandin E2 (PGL E2), leukotriene B4 (LT), 14,15-Epoxyeicosatrienoic acid (EET), 15-F2t-Isoprostane (IsoP), and resolvin D2 (bottom)), and acetylcarnitine (AC 2:0) and acylcarnitine (AC 18:0). Chemical structures are adapted from the LIPID MAPS structure database [25]. (**b**) Schematic overview of steps involved in the metabolism of fatty acyls, where fatty acids (FAs) can be obtained through the diet or by a multi-enzymatic reaction starting from acetyl-CoA and performed by enzymes such as acetyl-CoA carboxylase 1 (ACACA) and fatty acid synthase (FASN). Multiple steps of elongation, performed by elongases, and desaturation, carried out by desaturases, produce MUFA and PUFA. PUFA include, among others, omega-3 PUFA, such as ALA, which can be converted by a multistep reaction into eicosapentaenoic acid (EPA) and DHA, and omega-6 PUFA, including LA, which can be transformed by a multistep reaction to AA. PUFA can be further metabolized by enzymes such as lipoxygenase (LOX), prostaglandin-endoperoxide synthase 2 (PTGS2, also known as COX2), cytochrome p450 2C to various eicosanoids, including resolvins, PGL, LT, EET, or oxidized to isoP. Furthermore, transport of FA into mitochondria for their metabolism is preceded by their association with carnitine, which is catalyzed by the enzyme carnitine O-palmitoyltransferase 1 (CPT1) and reversed by carnitine O-palmitoyltransferase 2 (CPT2).

Fatty acyls are carboxylic acids formed by a hydrocarbon chain and a terminal carboxyl group (Figure 2) [25]. They are synthesized by chain elongation of acetyl-CoA with malonyl-CoA groups by enzymes named elongases. While humans can synthesize most fatty acyls, linoleic acid (LA) and alpha-linoleic acid (ALA) need to be obtained through the diet [26]. Fatty acyls are not only energy sources, but also the building blocks of complex lipids and as such form a key category of metabolites. Additionally, they are membrane constituents and regulate intracellular signaling, transcription factors, gene expression, bioactive lipid production, and inflammation [27,28]. Below, we will discuss the current knowledge of the roles of fatty acyls, more specifically of saturated fatty acids (SFA), monounsaturated fatty acids (MUFA), polyunsaturatedfatty acids (PUFA), eicosanoids and (acyl)carnitine, in PD, and an overview can be found in Supplementary Materials Table S1.

### 2.1. SFA

The simplest fatty acids are the straight-chain SFA. Their intake does not seem to be linked to PD risk in humans [29,30,31] per se, but SFA intake in individuals exposed to rotenone increases PD risk, when compared to pesticide exposure alone [32]. Thus, SFA could exacerbate PD-linked pathology. Interestingly, higher levels of SFA (mainly 16:0 and 18:0) have been observed in lipid rafts from the frontal cortex of PD patients compared to controls [33], but not in their temporal cortex [34]. These area-dependent changes combined with a lack of differences in SFA intake between PD patients and controls point to defects in their absorption or metabolism and region-specific and/or cell-compartment differences. Dietary supplementation with SFA 18:0, which seems to be a less potent pro-inflammatory lipid than other SFA species [35], regulates mitochondrial function and rescues the PD-like phenotype of *PINK* and *PRKN* mutant flies [36,37,38]. Similarly, both acute and repeated intra-gastric gavage of SFA 8:0 reduces the impairment of DA neurotransmission in MPTP-treated mice [39]. These findings, together with the observed higher SFA levels in the frontal cortical lipid rafts, may point towards a compensatory mechanism in PD patients. In contrast, exposure of SH-SY5Y cells, primary neurons, and astrocytes to SFA 16:0 leads to apoptosis, reduces peroxisome proliferator-activated receptor gamma coactivator 1-alpha (PPARGC1A, PGC-1alpha) and estrogen receptor alpha (ER-alpha) expression, promotes inflammation, and activates cyclooxygenase-2 (COX-2) [35,40,41,42], features that have also been observed in the brains of PD patients [43,44,45]. Since α-synuclein modulates the uptake of SFA 16:0 into the brain [46], accumulation of this protein in PD brains might lead to increased levels of SFA 16:0, which in turn can trigger some of its neuropathological activities.

### 2.2. MUFA

Variants of SFA containing one double bond are known as MUFA. Higher MUFA intake has been variably associated with decreased PD risk [31], reduced risk only in women [29] or unchanged risk [30]. These discrepancies in findings could be due to variation in the ethnicity of the subjects, differences in the type of study (cohort or case-control), the number of participants, questionnaires employed to asses MUFA intake, the corrections used, or even PD etiology, since different MUFA levels in cerebrospinal fluid (CSF) have been described in PD patients carrying a GBA mutation and those that do not [47]. Of note, no abnormalities in MUFA level have been observed in the temporal cortex of PD patients [34]. Some MUFA, such as oleic acid and cis-vaccenic acid, trigger the production of dopamine in MN9D cells [48], and the amide of oleic acid and dopamine (*N*-oleoyl-dopamine) modulates the firing of nigrostriatal DA neurons [49]. Interestingly, α-synuclein has a motif homologous to a region in fatty acid-binding proteins, allowing it to bind to oleic acid [50], which facilitates the interaction of α-synuclein with lipid rafts [51]. Based on these suggestive but still tentative findings, the effect of MUFA intake on PD risk, and specifically, its effect on dopamine production and the intracellular location and function of α-synuclein, should be further examined.

### 2.3. PUFA

Fatty acids containing two or more double bonds are known as PUFA and are usually classified according to the position of the first double bond counted from the tail (omega). The omega-3 family, for which ALA is the essential parent fatty acid, forms metabolic products that include eicosapentaenoic acid (EPA) and docosahexaenoic acid (DHA). Omega-6 PUFA, for which LA is the parent fatty acid, include arachidonic acid (AA), an intensely studied precursor of signaling lipids [52]. In general, PUFA are known to play a role in inflammation [53], epigenetics [54], and brain development [55] and function [56]. As such, they have been widely examined in PD patients, and animal and cellular PD models.

#### 2.3.1. Human Studies on PUFA

Higher intake of omega-3 PUFA and ALA, but not other PUFA, such as omega-6 PUFA or LA, has been associated with reduced risk of PD [31,32], while other studies have reported a weak positive association between omega-6 PUFA and LA intake and PD risk [57], or have provided evidence against an association between PUFA intake and PD risk [30]. A link between AA intake and PD risk is also controversial: one study described a positive association [30], while another reported an inverse association [58]. Serum of PD patients has decreased concentrations of long-chain PUFA, including ALA, LA, and AA, compared to controls [59], while the CSF of PD patients has increased levels of 4-hydroxynonenal, a toxic product generated by AA peroxidation [60]. However, as described for MUFA, PUFA levels in CSF may depend on PD etiology [47]. The levels of PUFA in the anterior cingulate cortex and the occipital cortex of PD patients are increased or not changed, respectively [61]. Additionally, the levels of DHA and AA are decreased in frontal cortex lipid rafts from PD patients [33], while reduced LA, increased DHA and docosatetraenoic acid (an omega-6 PUFA), and no changes in AA have been reported for the cytosolic fraction of PD frontal cortex [62]. Moreover, no changes of PUFA were observed in the temporal cortex of PD patients [34]. Therefore, there is no agreement on the impact of PUFA intake on PD risk and little information on PUFA levels in the blood, CSF and brain of PD patients is available. The only consistent finding is the altered intracellular distribution of PUFA in neurons from the frontal cortex of PD patients, i.e., reduced levels of DHA in the lipid rafts and increased DHA in the cytosolic fraction.

#### 2.3.2. Animal and Cellular Studies on PUFA

Omega-3 PUFA exert neuroprotective actions in MPTP-treated mice [63] by increasing the expression of brain-derived neurotrophic factor [64] and have also neuroprotective activity in 6-OHDA-treated rats [65]. A decrease in the level of this class of PUFA has been observed in the brains of an MPTP-induced goldfish PD model [66]. Furthermore, omega-3 PUFA deficiency leads to a reduced ability of the nigrostriatal system to maintain homeostasis under oxidative conditions, increasing the risk for PD [67]. Maternal omega-3 PUFA seem to partially protect a lipopolysaccharide (LPS)-model for PD [68]. Likewise, the omega-3 PUFA DHA protects DA neurons against MPTP–[69,70,71], paraquat—[72] or rotenone-induced toxicity [73] in rodent models and against effects of 6-OHDA-treatment in *Caenorhabditis elegans*, mice and rats [74,75,76], also when administered as TAG-DHA [77]. Moreover, DHA plays a crucial role in the differentiation of induced pluripotent stem cells (iPSCs) into functional DA neurons [78] and DHA supplementation protects DA neurons from the SN in MPTP-treated mice [79]. EPA and ethyl-EPA attenuate 1-methyl-4-phenylpyridinium (MPP+)-induced cell death in SH-SY5Y cells, primary mesencephalic neurons, and brain slices [80,81], and in vivo reduce MPTP/probecenid-induced dyskinesia and memory deficits (without preventing nigrostriatal DA loss) [82]. Thus, omega-3 PUFA appear to have a neuroprotective role in animal models for PD.

Additionally, pretreatment of rats with fish oil (which is rich in omega-3 PUFA) for 25 days before 6-OHDA treatment mitigates the loss of substantia nigra (SN) DA neurons [83]. In contrast, a chronic supplementation of fish oil in rats does not protect DA neurons but increases dopamine turnover [84]. These differential effects could be explained by the finding that the ethyl ester of DHA, a PUFA present in fish oil, enhances 6-OHDA-induced neuronal damage by triggering lipid peroxidation in mouse striatum [85]. Lipid peroxidation, which occurs frequently in PUFA, may lead to mitochondrial dysfunction [86] and α-synuclein oligomerization [87]. It is therefore not surprising that a number of studies have demonstrated beneficial effects when using deuterium-reinforced (deuterated) PUFA (which protects the PUFA sites susceptible for oxidation) [88,89,90], or PUFA in combination with antioxidants [91]. Therefore, omega-3 PUFA, probably in combination with the prevention of lipid peroxidation, should be further studied as a complementary therapy for PD.

Increased levels of omega-6 PUFA (LA and AA) have been reported in mice brain slices upon MPP+ treatment [81]. Similarly, upregulated AA signaling has been observed in the caudate-putamen and frontal cortex of 6-OHDA-treated rats [92], and the striatum and midbrain of MPTP-treated mice [93]. Both LA and AA are able to inhibit MPP+-induced toxicity in PC12 cells [94], while excess AA aggravates α-synuclein oligomerization in PC12 cells [95]. Interestingly, a mouse model with impaired incorporation of AA in the brain is resistant to MPTP treatment [96]. Hence, pharmacologically induced PD is linked to an increase in AA, the consequences of which are at present unclear and could be dose dependent.

#### 2.3.3. Alpha-Synuclein and PUFA

Under physiological conditions, α-synuclein and PUFA are involved in endocytic mechanisms linked to synaptic vesicle recycling upon neuronal stimulation [97]. Moreover, α-synuclein and PUFA regulate each other, since α-synuclein increases endogenous levels of AA and DHA [62], and its oligomers control the ability of AA to stimulate SNARE-complex formation and endocytosis [98]. Reciprocally, PUFA strongly interact with the N-terminal region of α-synuclein [99], enhancing its oligomerization both in vivo and in vitro [100,101,102]. This might precede the formation of protective (LB-like) inclusions in DA cells [103].

Studies on specific PUFA species have shown that DHA induces α-synuclein oligomerization [104] by activating retinoic X receptor and PPAR-gamma 2 [105], effects that were prevented by co-administering aspirin [106]. The oligomers formed in the presence of DHA seem to be cytotoxic [107] and affect membrane integrity [108] and the physical properties of DHA itself (triggering formation of lipid droplets) [109]. Alpha-synuclein aggregation is also induced by AA [110], but the oligomers that are formed seem to be less toxic (more prone to disaggregation and enzymatic digestion) [111] and their formation is prevented or enhanced by low or high doses of dopamine, respectively [112]. The enhanced toxicity of dopamine might be related to its ability to form adducts with AA, which are able to trigger apoptosis [113].

Interestingly, a diet poor in omega-3 PUFA (with or without DHA supplementation) did not affect α-synuclein expression [114]. Accordingly, a DHA-rich diet had no effect on the DA system, motor impairments or α-synuclein levels in α-synuclein-overexpressing mice but increased the longevity of the mice [115]. This latter phenomenon might be related to the role of monomeric α-synuclein in sequestering early DHA peroxidation products and thus reducing oxidative stress [116]. The interaction between α-synuclein and (peroxidated) PUFA has recently been reviewed in more detail elsewhere [87,117].

### 2.4. Eicosanoids and Docosanoids

Eicosanoids and docosanoids constitute a family of bioactive fatty acyls mainly generated by AA, EPA, and DHA oxidation. They play a local role in infection and inflammation [118]. The family includes PGL, LT, EET, isoprostanes, HETE, isofurans, and resolvins, among others. Interestingly, one of the enzymes responsible for the formation of eicosanoids, COX-2, has been linked to PD pathology. Its role in the disease has been reviewed elsewhere [119,120].

#### 2.4.1. PGL

No changes in or increased PGL E2 levels have been observed in the CSF and SN of PD patients, respectively [121,122]. In animal and cellular PD models, PGL E2 secretion is induced by LPS [123,124,125], 6-OHDA [126,127,128], rotenone [129,130], MPTP [131,132], and α-synuclein aggregation [133,134]. Nevertheless, PGL E2 levels in the striatum, hippocampus, and cortex of 6-OHDA-treated mice are decreased following a four-week exposure [135]. The eicosanoid PGL E2 mainly mediates its effects by binding to PGL E2 receptors (EP1-4), which trigger various intracellular pathways [136]. The EP1 receptor knock-out (KO) has neuroprotective effects on 6-OHDA-treated mice [137] and an EP1 antagonist protects embryonic rat mesencephalic primary cultures from 6-OHDA toxicity [138]. An agonist of EP2 protects primary neuronal cultures from 6-OHDA-induced toxicity [139] and an agonist of EP4 prevents DA loss in the SN of MPTP-treated mice [140]. Therefore, the effect of PGL E2 is also dependent on which receptor it binds. Interestingly, astrocytes KO for the familial PD gene *DJ-1* secrete less PGL E2 than WT astrocytes [141]. This could impair DA neuron survival mediated by EP2 [142]. Thus, the sparse data that are available regarding the effects of PGL suggest that increased PGL E2 levels may play a role in the pathology of animal and cellular PD models, but that this occurs in a time-, location-, phenotype- and receptor-dependent manner.

Both PGL A1 and lipocalin-type PGL D synthase (the enzyme that isomerizes PGL H2 to PGL D2) inhibit rotenone- and paraquat-induced apoptosis in SH-SY5Y cells, respectively [143,144]. Furthermore, enhanced prostacyclin synthesis seems to reduce glial activation and ameliorate motor dysfunction in 6-OHDA-treated rats [145]. Conversely, PGL J2 treatment of SK-N-SH cells leads to the formation of aggregates containing ubiquitinated α-synuclein [146], and infusion into the SN of mice induces a pathology that mimics the slow-onset cellular and behavioral pathology of PD, including loss of DA neurons in the SN, α-synuclein aggregation, posture impairment, and microgliosis [115,147,148]. Hence, PGL other than PGL E2 seem to play a role in PD pathology as well, with effects being protective or detrimental. To resolve this complexity and obtain deeper insight into the contributions of these oxidized PUFAs to PD pathology further research is needed.

#### 2.4.2. LT

Increased plasma LT B3 has been suggested as a biomarker for PD [149]. However, the role of LT has only been tested in animal and cellular PD models, in which MPTP treatment upregulates arachidonate 5-lipoxygenase (5-LOX, the enzyme that synthesizes LT from AA). This work has demonstrated that 5-LOX inhibition has neuroprotective effects [150], a finding which would be in agreement with the observation that LT B4 enhances MPP+-induced neurotoxicity in midbrain cultures [150]. Moreover, inhibition of cysteinyl LT receptor 1 has neuroprotective effects in a rotenone-induced rat PD model [151,152]. Interestingly, 5-LOX KO in mice reduces striatal dopamine levels under normal conditions [153]. Thus, 5-LOX seems to be necessary for maintaining the DA tone but can become deleterious upon toxicant challenge.

#### 2.4.3. EET

In PD patients, the SNP rs10889162 located in CYP2J2 (the enzyme that metabolizes AA into EET) is associated with age of diagnosis [154]. Interestingly, EETs are known to have cytoprotective effects in other diseases and may therefore also play a role in PD neuroinflammation [155]. In PD models, 14,15-EET, which is released from astrocytes, enhances cell viability against oxidative stress [156] and protects DA neuronal loss in MPTP-treated mice [157]. Inhibition or KO of the soluble epoxide hydrolase (sEH, inhibition of which elevates endogenous EET) protects MPTP-treated mice [157,158], and a double sEH and COX-2 inhibitor has protective effects on a rotenone-induced *Drosophila melanogaster* PD model [159]. Combined, these findings suggest that EET has widespread neuroprotective effects, not necessarily relevant for PD only.

#### 2.4.4. Isoprostanes

The role of isoprostanes in PD is controversial. Both higher levels and no change in F2-isoprostane have been found in urine and plasma [160,161,162] of (early) PD patients. Moreover, no changes have been observed in CSF [163] or SN [164] of PD patients, but higher levels of F2-isoprostane have been described in anterior cingulate cortex of PD patients [61]. Higher F2-isoprostane levels have also been observed in rotenone-, but not manganese-treated DA neurons derived from healthy iPSC [165]. Thus, more research and proper stratification of findings need to be performed to understand the role of isoprostanes in PD.

#### 2.4.5. Other Eicosanoids and Docosanoids

The classic and non-classic AA-derived eicosanoids, HETE and isofurans, are increased in plasma [161,162] and SN [164] of PD patients, respectively. The docosanoid resolvin D1 attenuates MPP+-induced PD by inhibiting inflammation in PC12 cells [166], and resolvin D2 seems to restore LPS-induced neural injury in a rat model, also by suppression of inflammation [167]. Thus, resolvins seem to have a protective role in PD.

### 2.5. Carnitine and Acylcarnitine

Carnitine is a trimethyllysine derivative that can associate with various fatty acids, forming acylcarnitine. This association facilitates their transport from the cytosol to the mitochondrial matrix, where fatty acids undergo β-oxidation. Both carnitine and acylcarnitines are involved in processes such as neurotransmission and apoptosis [168]. Decreased levels of carnitine and (long-chain) acylcarnitines have been detected in plasma from PD patients [149,169,170], while no changes in acylcarnitine levels have been found in either CSF or plasma from PD patients when compared to controls [171]. Acetylcarnitine (acylcarnitine 2:0) protects SK-N-MC cells from rotenone-induced toxicity [172], and carnitine reduces the effects of MPP+ on rat forebrain primary cultures [173] and LPS in SIM-A9 microglial cells [174]. Moreover, the neuroprotective properties of acetylcarnitine have been found in rats treated with 6-OHDA [175,176,177] and rotenone [178,179], and non-human primates treated with MPTP [180]. Additionally, increased levels of carnitine and acylcarnitine 16:0 and 18:0 have been detected in the striatum of 6-OHDA-treated rats and the mesencephalon of MPTP-treated mice, respectively [181,182], suggesting a compensatory mechanism against PD-associated toxicity. Thus, while it remains unclear whether the levels of acylcarnitine change in PD patients, mounting evidence points towards decreased plasma levels and a protective role of acylcarnitine in animal and cellular PD models.

## 3. Glycerolipids

**Figure 3:**
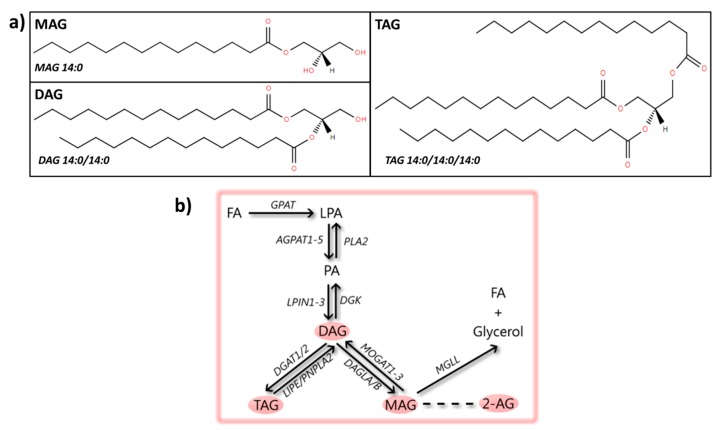
Glycerolipids: structures and metabolic steps involved. (**a**) Schematic representation of the chemical structures of glycerolipids, including monoacylglycerol (MAG 14:0), diacylglycerol (DAG 14:0/14:0), and triacylglycerol (TAG 14:0/14:0/14:0). Chemical structures are adapted from the LIPID MAPS structure database [25]. (**b**) Schematic overview of metabolic steps involved in the synthesis and conversion of glycerolipids: synthesis starts from fatty acids (FAs) by sequential conversion into LPA and PA, which are phospholipids (process described in Figure 4b). The enzyme phosphatide phosphatase (LPIN1-3) converts PA into DAG, a step that can be reversed by diacylglycerol kinase (DGK). From DAG, one FA can be added to the glycerol backbone by diacylglycerol O-acyltransferase 1/2 (DGAT1/2), creating TAG, a step that can be reversed by the hormone-sensitive lipase (LIPE) or patatin-like phospholipase domain-containing protein 2 (PNPLA2). Additionally, one FA can be removed from DAG by the enzyme sn1-specific diacylglycerol lipase alpha/beta (DAGLA/B), giving rise to MAG, which can be transformed back to DAG by 2-acylglycerol O-acyltransferase 1-3 (MOGAT1-3). One of the mostly studied MAG species is the endocannabinoid 2-arachidonoylglycerol (2-AG). MAG can be degraded to glycerol and a FA by monoglyceride lipase (MGLL).

The esterification of one, two or three fatty acyls with glycerol gives rise to the glycerolipids mono-, di-, and tri-substituted glycerol, known as monoacylglycerol (MAG), diacylglycerol (DAG), and triacylglycerol (TAG), respectively (Figure 3). There is little information available on the function of MAG, while DAG is a neutral lipid involved in the formation of membranes [55] and in the synaptic vesicle cycle [183]. Additionally, DAG fulfills a role as secondary lipid messenger [184]. The neutral lipid TAG is the main energy storage molecule [185]. Below, we will discuss the current knowledge of the roles of all glycerolipids with potential significance for PD, including MAG, endocannabinoids, DAG, and TAG, and an overview is given in Supplementary Materials Table S1.

### 3.1. MAG

Decreased and increased expression of MAG lipase, the enzyme that degrades MAG to glycerol and free fatty acids, has been observed in the SN and the putamen of PD patients, respectively [186]. These differential effects could be associated with the different mechanisms leading to the degeneration of these brain areas; in PD patients, the SN presents with cell loss [187], while the putamen has DA depletion [188]. In PD models, pharmacological inhibition of MAG lipase has neuroprotective effects in both SH-SY5Y cells treated with MPP+ [189] and chronic MPTP/probenecid mouse models [190,191]. Although there is no information on the levels of MAG in PD patients, the model studies suggest that MAG lipase inhibition, and thus higher levels of MAG, may be protective for PD.

#### Endocannabinoids

MAG include 2-arachidonoylglycerol (2-AG), which is classified as an endocannabinoid. Endocannabinoids are a heterogeneous and thus difficult to classify group of lipids, including not only 2-AG, but also fatty acyl amides, such as anandamide (AEA). They have been previously linked to PD [192].

Two studies have reported high AEA levels in the CSF of untreated PD patients, which were restored upon DA treatment [193,194]. Moreover, higher cannabinoid 1 receptor (CB1R) levels in the putamen, and higher and lower cannabinoid 2 receptor (CB2R) levels have been reported in the SN and putamen of PD patients, respectively [186]. Treatment of PD patients displaying no psychiatric comorbidities with cannabidiol, a naturally occurring cannabinoid constituent of cannabis which appears to lack psychoactive effects, improves quality of life measures, but does not improve Unified Parkinson’s Disease Rating Scale scores [195]. The toxin 6-OHDA has been found to increase CB1R mRNA expression [196], downregulate CB1R protein density in multiple brain regions [197,198] or not produce any changes [199]. These differential effects may be explained by the fact that 6-OHDA seems to change the expression of CB1R protein in a region- and time-specific manner [200]. Decreased CB1R mRNA expression has been described in the striatum of reserpine-treated rats [201] and increased CB1R protein density has been observed in *PRKN* KO female mice [202]. Moreover, CB1R agonists fail to modulate spontaneous excitatory postsynaptic currents in cortical synapses of PINK1 KO mice [203], which points towards a CB1R dysfunction in these synapses. Hence, there is no agreement on the modulation of CB1R in different PD models and its correlation with the pathogenesis of PD in humans, making further studies necessary.

Drug-induced animal PD models (using rotenone, 6-OHDA or LPS) show increased CB2R mRNA expression [204,205], while a genetic model for PD (*LRRK2* KO) does not display changes in CB2R mRNA levels [206]. However, CB2R agonists appear to improve PD-linked impairments in both drug- and genetically-induced rodent PD models [206,207,208]. Thus, CB2R upregulation in animal models may reflect a compensatory mechanism, since the administration of CB2R agonists has positive effects on PD-linked pathology.

Reduced levels of the AEA precursor synthesizing enzyme, *N*-acyl-transferase [209], and reduced activities of the AEA membrane transporter and hydrolase [210,211] have been observed in the striatum of 6-OHDA-treated rats. Moreover, 6-OHDA treatment has been found to both decrease [212] and increase [210,211] striatal levels of AEA, while MPTP-lesions in monkeys increase striatal AEA levels [213]. Interestingly, an increase of AEA levels, by inhibition of the fatty acid amide hydrolase or administration of AM404 (an endogenous cannabinoid reuptake inhibitor), has neuroprotective effects [209,210,214,215]. Hence, AEA seems to have neuroprotective effects, which have been suggested to be mediated by activation of PI3K and inhibition of JNK signaling [216].

All MPTP, rotenone, and reserpine treatments lead to increased 2-AG levels in a time- and region-specific manner in various animal PD models [213,217,218,219], and 2-AG administration provides protection against MPTP-induced cell death [217]. The endocannabinoid *N*-arahidonoyl-dopamine has anti-inflammatory effects on both macrophages and activated BV-2 cells [96] and modulates the activity of SN neurons [220], together with the endocannabinoid-like *N*-oleoyl-dopamine [49]. This effect could be linked to the fact that, together with an inhibitor of endocannabinoid degradation, administration of a D2 receptor agonist improves motor performance in both a 6-OHDA-and a reserpine-model for PD [221]. Similar to AEA, different members of the endocannabinoid group of lipids may thus be neuroprotective.

### 3.2. DAG

Parkinson’s disease patients have decreased plasma levels of DAG [222,223], and increased DAG levels in frontal cortex [224] and primary visual cortex [225]. Interestingly, SNPs from the chromosomal region that includes the gene encoding diacylglycerol kinase theta (*DGKQ*), which mediates the production of phosphatidic acid (PA) from DAG, are associated with PD susceptibility [14,226,227], and *DGKQ* is linked to increased PA 36:2 production and consequent α-synuclein aggregation [228]. The dysregulation of integral DAG metabolism in PD patients could be related to the observed genetic association between *DGKQ* and PD.

### 3.3. TAG

TAG levels are decreased in serum and plasma of (male) PD patients [222,223,229,230,231,232,233], even before diagnosis [234], and higher serum TAG is associated with reduced risk of idiopathic PD [235]. However, other studies have found no differences in blood TAG levels of PD patients and controls [236,237]. In the primary visual cortex of PD patients, the levels of TAG are decreased [225]. Thus, reduced levels of TAG seem to be linked to PD, although high heterogeneity has been described for TAG in PD patients [238]. Gender, ethnicity, or the technique used to measure TAG could bias the obtained results and contribute to the observed heterogeneity. Nevertheless, the trend from the majority of the results is in line with findings in animal models for PD, in which α-synuclein A53T overexpression leads to deceased serum TAG levels [239], and 6-OHDA treatment decreases TAG levels in retroperitoneal white adipose tissue [240]. Both rotenone and α-synuclein overexpression have been linked to intracellular deposition of TAG [241,242], which forms lipid droplets to which α-synuclein binds, and as such, the turnover of stored TAG is reduced and α-synuclein aggregation is enhanced [243]. In agreement, α-synuclein A53T overexpression in N27 cells leads to increased intracellular levels of TAG [244]. Thus, intracellular deposition and reduced turnover of TAG may explain the reduced levels of this acylglycerol in PD serum. Interestingly, *Saccharomyces cereviciae* that are unable to synthesize TAG are more tolerant to α-synuclein overexpression [242].

## 4. Glycerophospholipids

**Figure 4:**
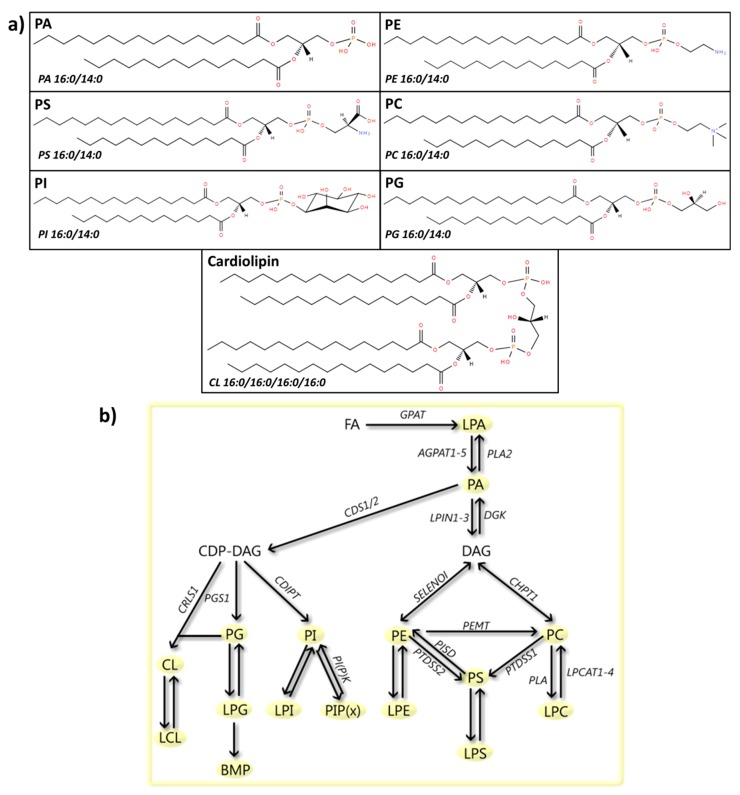
Phospholipids: structures and metabolic steps involved. (**a**) Schematic representation of the chemical structures of phospholipids, including phosphatidic acid (PA 16:0/14:0), phosphatidylethanolamine (PE 16:0/14:0), phosphatidylserine (PS 16:0/14:0), phosphatidylcholine (PC 16:0/14:0), phosphatidylinositol (PI 16:0/14:0), phosphatidylglycerol (PG 16:0/14:0), and cardiolipin (CL 16:0/16:0/16:0/16:0). Chemical structures are adapted from the LIPID MAPS structure database [25]. (**b**) Schematic overview of phospholipid metabolism. Synthesis starts with the conversion of fatty acids into lysophosphatidic acid (LPA) by glycerol-3-phosphate acyltransferase (GPAT). LPA is then metabolized to PA by 1-acyl-sn-glycerol-3-phosphate acyltransferase 1-5 (AGPAT1-5), a reaction that can be reversed by phospholipase A2 (PLA2). PA can then be metabolized to diacylglycerol (DAG) (process described in Figure 3b), which can be subsequently transformed to PE by ethanolaminephosphotransferase 1 (SELENOI) or PC by cholinephosphotransferase 1 (CHPT1). PE can also be converted into PC by the enzyme phosphatidylethanolamine *N*-methyltransferase (PEMT). Both compounds can be precursors for the synthesis of PS by the enzymes phosphatidylserine synthase1/2 (PTDSS1/2). The conversion of PE to PS can be reversed by phosphatidylserine decarboxylase proenzyme (PISD). Additionally, PA can be metabolized by phosphatidate cytidylyltransferase 1/2 (CDS1/2) to cytidine diphosphate DAG (CDP-DAG), which can then be transformed to either PI, PG or CL, by CDP-diacylglycerol--inositol 3-phosphatidyltransferase (CDIPT), CDP-diacylglycerol--glycerol-3-phosphate 3-phosphatidyltransferase (PGS1) and cardiolipin synthase (CRLS1), respectively. PI can be phosphorylated by PI (phosphate) kinases (PI(P)K), to produce PI phosphate (PIP(x)). Moreover, all phospholipids can be metabolized to their lyso-forms (LPC, LPS, LPE, LPI, LPG, and LCL) by PLA2, a reaction reversed by lysophospholipid acyltransferases (LPCATs). LPG can be further metabolized to Bis(monoacylglycero)phosphate (BMP).

Glycerophospholipids, or phospholipids, have a glycerol backbone and a polar head group, which allows their classification into distinct subgroups, known as PA, phosphatidylethanolamine (PE), phosphatidylserine (PS), phosphatidylcholine (PC), phosphatidylinositol (PI), phosphatidyllycerol (PG) and cardiolipin (CL) (Figure 4). The hydrolysis of one acyl derivative gives rise to the lipid species known as lysophospholipids. Glycerophospholipids are key components of the lipid bilayers of cells, and as such play a role in organelle function [245] and processes like endocytosis [246] or mitophagy [247]. Moreover, they also act as signaling molecules [248,249,250] and regulate lipid metabolism-related gene expression [251]. Below, we will discuss the current research on glycerophospholipids in PD, more specifically PA, PE, PS, PC, PI, PG and CL, and an overview is given in Supplementary Materials Table S1.

### 4.1. PA

One of the best-known glycerophospholipid messengers is PA, which has a broad spectrum of functions, including intracellular vesicular trafficking, cell survival, cytoskeletal organization, neuronal development, and mitochondrial function [252,253,254]. Increased plasma PA (18:2/15:0) levels have been suggested as a biomarker for PD [149]. Additionally, PA is known to interact with residues 1–102 of α-synuclein [255,256], thus enhancing the formation of multimeric and protease-resistant α-synuclein aggregates [257,258]. ATP13A2, a lysosomal ATPase, which, when mutated, causes familial PD, constitutes another link between PA and PD. This ATPase requires the interaction with PA, and also PI(3,5)P2, to protect cells against rotenone-induced mitochondrial stress or other PD-related stress conditions, such as exposure to Fe(3+) [259,260]. Furthermore, overexpression of phospholipase D2 appears to induce DA neuronal cell loss via a mechanism involving PA signaling [261]. Given PA’s role in the subcellular distribution and aggregation of α-synuclein, and in ATP13A2-mediated neuroprotection, it would be of interest to study PA levels and its partitioning in the brains of PD patients.

#### LPA

Similar to PA, LPA is a lipid mediator in a wide range of biological actions, including cell proliferation, (nervous system) development, and cytokine secretion [262,263,264]. Furthermore, LPA is involved in neuronal (DA) differentiation [265]. The expression of LPA receptor 1 is reduced in the SN of a 6-OHDA rat PD model [265], and an LPA receptor ligand attenuates the MPTP mouse PD model [266]. Unfortunately, nothing is known about LPA in PD patients.

### 4.2. PE

The glycerophospholipid PE has a structural role in biological membranes, and it is a regulator of cell division, membrane fusion/fission, and hepatic secretion of very low-density lipoproteins (VLDL) [267]. Patients with PD show decreased plasma levels of PE 34:2 [222], and those carrying a *GBA* mutation have decreased serum levels of PE compared to non-*GBA* mutation carriers [268]. Decreased total PE levels have also been observed in the SN of PD patients before treatment [269], in males only after treatment [270], and in the primary visual cortex [225]. In contrast, increased PE has been found in frontal cortex lipid rafts from PD patients [33]. Of note, one of the enzymes linked to PE synthesis, phosphoethanolamine cytidylyltransferase, is elevated in the SN of PD patients [271]. All findings combined, most evidence points towards decreased levels of PE in PD patients, but the biological implications of the reduced levels need to be examined further.

In vitro, PE is necessary for the interaction between α-synuclein and biological membranes [272] and for the formation of stable, highly conductive channels by α-synuclein [273]. Both processes might have a role in the normal function of α-synuclein. Accordingly, in yeast and worm models, PE deficiency disrupts α-synuclein homeostasis and induces its aggregation [274,275]. This deficiency, also seen in PD patients, could be due to increased formation of LPC from PE, which occurs in MPP+ models [276]. Moreover, the inhibition of this metabolic step offers significant protection against cytotoxicity [277].

### 4.3. PS

The glycerophospholipid PE is involved in the triggering of both intracellular and extracellular cascades, such as the activation of kinases or the clearance of apoptotic cells [250,278]. It plays a role in neuronal survival and differentiation, and neurotransmitter release [279]. Plasma levels of PS 40:4 are decreased in PD patients [222], but higher levels of PS 36:1, PS 36:1, 36:2, and 38:3, or overall PS, have been found in parkin-mutant fibroblasts [280], frontal cortex [224], and primary visual cortex [225] of PD patients, respectively. This is in agreement with the increased PS synthase activity that has been observed in the SN of PD patients [271]. However, some groups reported contrasting findings and claimed that total PS levels in PD SN and frontal cortex lipid rafts are not significantly altered [33,270]. Yet another interesting finding is that parkin-mutant iPSC-derived neurons have a different subcellular distribution of PS [281], with increased and decreased PS in the mitochondrial and ER fractions, respectively.

The exposure of PS on the cellular surface, which acts as an “eat-me” signal for phagocytosis, is triggered by 6-OHDA [282], rotenone [283], paraquat [284], MPP+ [285], and WT, A53T and A30P α-synuclein [286]. Blockade by an antibody against PS is protective in a rotenone-induced neuronal/glial PD model [287], pointing towards a role of microglial-mediated phagocytosis in PD. This glyceroplipid is known to be associated with the N-terminal- and mid-region of α-synuclein [256,288], with some preference for acetylated α-synuclein [289,290]. This association correlates with membrane penetration [255], alpha-helix formation [256] and aggregation [291], and vesicle [256,292] and liposome [51] binding. Taken together, these findings suggest that PS is a modulator of apoptosis and α-synuclein-mediated pathology.

### 4.4. PC

The most-abundant glycerophospholipid in eukaryotic membranes, including mitochondrial membranes [293], where it plays a structural role, is PC. It is involved in anti-inflammation [294], cholesterol metabolism [295], and neuronal differentiation [296]. Decreased levels of PC 34:2 and 46:2, PC 34:5, 36:5, and 38:5, and total PC, have been observed in plasma and frontal cortex from PD patients [222,224], and in SN from only male PD patients [270], respectively. One of the enzymes involved in PC synthesis, PC cytidylyltransferase, is elevated in the SN of PD patients [271]. Interestingly, components of the pathway “PC biosynthesis”, together with “PPAR signaling” components, allowed accurate classification of PD and control samples [297], highlighting altered PC metabolism as a consistent feature of PD.

Decreased PC levels have been found in the SN of a mouse model of early PD [298] and in brain tissue from MPTP-treated goldfish [66]. Interestingly, α-synuclein does not bind to but rather remodels pure PC membranes through weak interactions with this phospholipid [299,300], and α-synuclein E46K mutants form functionally distinct ion channels in PC membranes [301]. However, others observed binding of the physiologically relevant N-terminally acetylated α-synuclein to pure PC membranes, with preference for highly curved and ordered membranes [302]. Based on these multiple links, the significance of PC metabolism for PD pathology is an interesting and important topic for further study.

#### LPC

The most-abundant lysophospholipid in the blood is LPC. Its levels are critically related to major alterations in mitochondrial function (e.g., oxidation rate) and to minor defects in mitochondrial permeability [303,304]. Of note, saturated acyl LPCs have inflammatory properties, such as leukocyte extravasation and formation of pro-inflammatory mediators, which can be compensated by polyunsaturated acyl LPC, such as LPC 20:4 and LPC 22:6 [305]. Higher levels of LPC 16:0 and 18:1 have been found in the lipid profile of parkin-mutant fibroblasts compared to healthy controls [280]. Moreover, increased plasma LPC 18:2 has been suggested as a biomarker for PD [149].

Treatment with MPTP induces LPC formation, which leads to cytotoxic changes, dopamine release and inhibition of its uptake, a decreased mitochondrial potential, and increased reactive oxygen species (ROS) formation in PC12 cells [277]. The lysophospholipid LPC inhibits D1 and D2 receptor binding activities in the striatum of rats, inhibits the dopamine transporter, and decreases striatal dopamine turnover rate [306], leading to hypokinesia [307]. Interestingly, 6-OHDA treatment of rats gives rise to an overall decrease in LPC species, with the exception of LPC 16:0 and 18:1, which are increased in the SN [298]. Thus, LPC has negative effects on the DA system, but LPC levels in PD models seem to depend on the type of pharmacological treatment used and the LPC species involved.

### 4.5. PI

The glycerophospholipids PI and PI phosphates are part of intracellular signal transduction systems [308], but relatively little is known about their role in PD. In humans, higher levels of PI 34:1 and no changes of PI 36:1, 36:2, 38:4, 38:5, 40:5, and 40:6, or no changes in total PI have been observed in parkin-mutant skin fibroblasts [280], and in the lipid rafts of frontal cortex from PD patients [33], respectively. Decreased levels of overall PI have been observed in the SN of male PD patients [270]. In rodents, MPTP decreases the expression level of striatal PI-transfer protein [309], which is involved in the transfer of PI across membranes [310].

#### PI Phosphate (PIPx)

The role of PIPx species in PD is also poorly defined. However, PI and PIP2 effectively influence self-oligomerization of α-synuclein [311], while α-synuclein seems to prefer binding membranes containing PI(4,5)P2 [312]. Moreover, PIP3 is decreased in the nuclear fraction and whole-tissue homogenate, while PIP2 is increased in whole-tissue homogenate of SN from PD patients [313]. As mentioned above, ATP13A2 requires the interaction with PI(3,5)P2 to protect cells against PD-related stress conditions [259,260], an interaction that is able to reduce proteasomal inhibitor-induced accumulation of ubiquitin proteins [314]. Future systematic studies on the roles of the various PIPx species are required to understand their function in PD pathogenesis.

### 4.6. PG

Less than 1% of total glycerophospholipids in intracellular membranes is composed of PG and it is mainly localized to mitochondrial membranes, where it can be synthesized locally [315]. The levels of total PG are not changed in lipid rafts from PD frontal cortex [33], while increased PG 32:0 has been described in total extracts of the same brain area [224]. Alpha-synuclein is able to bind PG with various degrees of affinity depending on the variability in its structure (WT ≈ truncated > A53T > A30P) [316], and PG-containing membranes can promote α-synuclein aggregation [317,318,319]. Additionally, α-synuclein oligomers are able to induce PG clustering [319], connect PG-containing vesicles [320] and disrupt PG vesicles [321] through large membrane bilayer defects, rather than through a pore-like mechanism [322], leading to vesicle docking and fusion problems. Furthermore, low concentrations of α-synuclein inhibit and high concentrations stimulate lipid peroxidation of PG [323]. Unfortunately, information on the levels of PG in animal PD models is lacking.

### 4.7. CL

The glycerophospholipid specific for mitochondrial membranes is CL. Here, it plays both a structural and functional role [324,325,326]. No changes in total CL levels have been detected in the SN of PD patients [270]. However, *PINK1* KO mouse embryonic fibroblasts display decreased CL levels and supplementation with CL rescues mitochondrial dysfunction [327]. Moreover, rotenone induces oxidation of highly unsaturated CL in human peripheral blood lymphocytes [328], and increases levels of plasma PUFA CLs, but decreases oxidizable PUFA-containing CL levels and increases mono-oxygenated CL species in the SN of rats [329]. A proper CL content in the inner mitochondrial membrane and the presence of acyl side chains are crucial for α-synuclein localization [330,331], while CL content in the outer mitochondrial membrane buffers synucleinopathy [332]. Moreover, α-synuclein is able to disrupt artificial membranes containing CL [333], and its overexpression reduces CL content in MN9D cells [334] and in mouse brain [335]. Additionally, the formation of complexes between CL and α-synuclein, together with cytochrome c, may be a source of oxidative stress [336]. The role that CL plays in the interaction of α-synuclein with membranes and in mitophagy has been previously reviewed [337,338].

## 5. Sphingolipids

**Figure 5:**
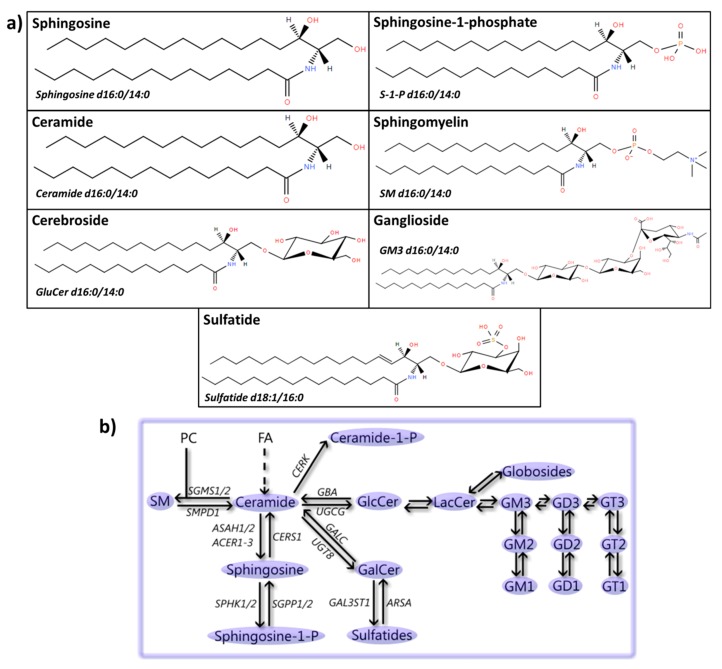
Sphingolipids: structures and metabolic steps involved. (**a**) Schematic representation of the chemical structures of sphingolipids, including sphingosine (d16:0/14:0), sphingosine-1-phosphate (S-1-P d16:0/14:0), ceramide (d16:0/14:0), sphingomyelin (SM d16:0/14:0), cerebroside (glucosylceramide, GluCer, d16:0/14:0), ganglioside (GM3 d16:0/14:0), and sulfatide (d18:1/16:0). Chemical structures are adapted from the LIPID MAPS structure database [25]. (**b**) Schematic overview of steps involved in the formation and metabolic conversion of sphingolipids. Synthesis of sphingolipids starts by a multistep process to convert fatty acids (FAs) into ceramide. Phosphatidylcholine can be fused to ceramide by phosphatidylcholine:ceramide cholinephosphotransferase 1/2 (SGMS1/2) to produce SM, which can be converted back into ceramide by sphingomyelin phosphodiesterase (SMPD1). Ceramide can also be phosphorylated by ceramide kinase (CERK) to ceramide-1-P and converted into sphingosine by acid or alkaline ceramidases (ASAH1/2 or ACER1-3), and phosphorylated by sphingosine kinase 1/2 (SPHK1/2) to sphingosine-1-phosphate. This process can be reversed by the sequential action of sphingosine-1-phosphate phosphatase 1/2 (SGPP1/2) and ceramide synthase 1 (CERS1). Furthermore, ceramide can be glycosylated via the addition of a galactose molecule by 2-hydroxyacylsphingosine 1-beta-galactosyltransferase (UGT8) to produce galactosylceramide (GalCer). Further addition of a sulfate group by galactosylceramide sulfotransferase (GAL3ST1) results in the formation of sulfatides. This process can be reversed by the sequential actions of arylsulfatase A (ARSA) and galactocerebrosidase (GALC). Finally, ceramide can also by glycosylated via the addition of a glucose molecule by ceramide glucosyltransferase (UGCG) to produce GlcCer, which can be further glycosylated to produce both globosides and gangliosides, such as GM3, GD2 and GT1, by multiple enzymes [340], or it can be converted back into ceramide by glucosylceramidase (GBA).

Sphingolipids constitute a family of lipids characterized by the presence of a sphingoid-base backbone. This complex family of compounds includes the sphingoid bases (e.g., sphingosine and sphingosine-1-phosphate), ceramides, phosphosphingolipids (e.g., sphingomyelin (SM)) and glycosphingolipids (e.g., cerebrosides, ganglisodes, and sulfatides) (Figure 5). Sphingolipids are not only structural components of cell membranes, but they also play a role in apoptosis, autophagy, and immune response [339]. Here, we will specifically focus on the involvement of sphingosine(-1-phosphate), ceramide, SM, cerebrosides, gangliosides, and sulfatides, and an overview is given in Supplementary Materials Table S1.

### 5.1. Sphingosine(-1-Phosphate)

Sphingosine is a bioactive lipid known to induce apoptosis and regulate endocytosis, while its phosphorylated form, sphingosine-1-phosphate (S1P), promotes cell survival and triggers diverse intracellular signaling pathways through G-protein-coupled receptors [339,341,342]. Sphingosine induces the formation of oligomeric α-synuclein species, which serve as template for the formation of endogenous α-synuclein aggregates in human and mammalian neurons [343]. Similarly, S1P accumulation, e.g., due to GBA deficiency, promotes α-synuclein aggregation [343]. Alpha-synuclein itself inhibits the expression and activity of sphingosine kinase 1, the enzyme that catalyzes the phosphorylation of sphingosine to S1P [344] and modulates S1P receptor-mediated signaling [345,346]. Sphingosine-1-phosphate supplementation of MPP+-treated cells is neuroprotective [347,348,349], and a selective S1P receptor agonist is protective in mouse and cellular models treated with 6-OHDA and rotenone [350]. Therefore, while S1P is protective in animal and cellular PD models, presumably through its pro-survival effects, it is clear that both sphingosine and S1P are linked to α-synuclein aggregation. Unfortunately, the lack of studies on human samples does not allow drawing a conclusion regarding the relevance of these lipids for PD pathogenesis.

### 5.2. Ceramide

Ceramide is involved in apoptosis, lipid raft formation, and regulation of the mitochondrial respiratory chain [340,351,352,353]. Both higher [354] and lower [222,355] plasma levels of ceramide have been reported in PD patients, while lower ceramide 18:0 and no differences in total ceramide levels are observed in their frontal cortex [224] and SN [270], respectively. Reduced levels of ceramide may be associated with α-synuclein accumulation [356,357]. This is in line with the finding that reduced and increased levels of ceramide have been observed in the anterior cingulate cortex and primary visual cortex of PD patients [225,356,358], which display and lack α-synuclein aggregation, respectively [359]. Thus, variation in the levels of ceramide in different tissues may be linked to α-synuclein accumulation.

Mimicking PD with *PLA2G6* KO, *LRRK2* KO, *PINK1* KO or rotenone treatment increases ceramide levels in fly brain, mouse brain, mouse olfactory bulb, and human erythrocytes, respectively [283,360,361,362]. C2-ceramide initiates a series of events leading to neuronal death, including an early inactivation of PI3K/AKT and ERK pathways, followed by activation of JNK, GSK3β activation and neuronal death [363]. Additionally, C2-ceramide induces cytotoxicity and ROS production in neuronal(-like) cells [364,365,366], which can be prevented by WT α-synuclein [367], *PINK1* [368,369] and *DJ-1* [370]. However, both in vivo and in vitro C2-ceramide seems to suppress microglial activation [371], protect neurons against α-synuclein-induced cell injury [372], and reverse rotenone-induced phosphorylation and aggregation of α-synuclein [373]. An increase in ceramide levels is thus commonly found in animal and cellular models for PD, but its effects are unclear and may be both beneficial and detrimental for different PD-related traits.

### 5.3. SM

The most abundant sphingolipid in eukaryotic cells and plasma is SM. It is one of the building blocks of the cellular membrane and a source of bioactive lipids, such as ceramide, ceramide-1-phosphate and S1P, which are involved in inflammation [374,375], cell death [376,377] and autophagy [378]. In the nervous system, SM is a major constituent of myelin. Mutations in sphingomyelinase-1, which lead to SM accumulation, are a risk factor for PD [16,379,380]. This feature may be linked to the increase in α-synuclein expression observed upon SM treatment [381] and the presence of SM in LB inclusions [382]. Parkinson’s disease patients carrying GBA mutations have elevated levels of total plasma SM compared to PD patients not carrying the mutation [268]. Moreover, SM 18:1 and SM 26:1 are increased and decreased in the anterior cingulate cortex [358], respectively, while increased SM levels have been described in the primary visual cortex [225], and, in males only, in the SN of PD patients [270]. However, no changes have been found in the putamen or cerebellum of sporadic PD patients [383]. The role that SM accumulation appears to play in PD pathogenesis may thus be multifold, being linked to inflammation, autophagy dysfunction, and/or α-synuclein expression and aggregation.

### 5.4. Cerebrosides

Cerebrosides are lipids glycosylated via the addition of either glucose or galactose and known to be involved in intracellular membrane transport and cell survival [384]. In PD patients, cerebrosides are increased in plasma (of *GBA* mutation carriers) and, in males only, in the SN, whereas they are decreased in lipid rafts from the frontal cortex [33,268,270,385]. More specifically, PD patients have increased levels of glucosylceramide [223,354] in plasma but no changes in cerebroside levels in the temporal cortex [386], putamen or cerebellum [383], and decreased levels of galactosylceramide 24:1 and lactosylceramide 18:1 in the frontal cortex [224]. Thus, whereas a consistent coupling between PD and increased cerebrosides in plasma has been found, cerebroside changes in the brain are region dependent and their significance for PD needs to be determined.

Interestingly, mutations in the enzymes responsible for the degradation of cerebrosoides, namely GBA and galactocerebosides (GALC), which cause Gaucher’s disease and Krabbe’s disease, respectively, have been associated with α-synuclein aggregation and PD [387,388]. Glucosylceramide, a product that accumulates upon GBA deficiency, destabilizes α-synuclein tetramers and related multimers and frees α-synuclein monomers and leads to cellular toxicity [389]. These effects are caused by colocalization of glucosylceramide with α-synuclein and induction of a pathogenic conformational change of the protein [390]. This promotes aggregation of WT (but not mutated) α-synuclein into a β-sheeted conformation [343,391], and conversion of α-synuclein into a proteinase-resistant form [392]. Conversely, α-synuclein inhibits normal activity of GBA [393], which increases glucosylceramide, creating a feedback loop. Inhibition of glucosylceramide synthase, which decreases glucosylceramide levels, slows α-synuclein accumulation [394] and partially protects mice against MPTP-induced toxicity [395]. Thus, it is well established that glucosylceramide accumulation leads to α-synuclein aggregation and toxicity. Interestingly, aging of WT mice leads to brain accumulation of both glucosylceramide and lactosylceramide [396], suggesting that age-associated changes in its metabolism might be related to PD onset.

### 5.5. Gangliosides

Gangliosides are synthesized by the addition of carbohydrate moieties to lactosylceramide. One of the simplest and most widely distributed ganglioside is monosialodihexosylganglioside (GM3) that consists of lactosylceramide and sialic acid [397]. Gangliosides were initially discovered in the brain where they are involved in neurotransmission, receptor regulation, and stabilization of neural circuits, including the nigro-striatal DA pathway [398,399]. Parkinson’s disease patients have higher plasma levels of gangliosides [385], GM3 gangliosides [223], and N-acetylneuraminic acid-3 (NANA-3) gangliosides [222] than controls. Likewise, higher GM2 and GM3 levels have been detected in parkin-mutant iPSCs compared to controls [280]. However, no accumulation of GM1, GM2 or GM3 has been observed in the putamen or cerebellum of sporadic (or heterozygous GBA-mutation) PD patients [383], nor in the SN of PD patients [270]. Even a GM1 deficiency, together with decreased expression of ganglioside biosynthetic enzymes (B3GALT4 and ST3GAL2), has been found in the SN from PD patients [400,401]. Hence, most publications point towards increased gangliosides in plasma of PD patients, but concomitant changes in ganglioside levels have not been observed in their brains.

Interestingly, GM1 supplementation seems to have a positive disease-modifying effect in PD patients [402,403,404,405,406,407]. Also, increased GM1 levels are neuroprotective in MPTP-treated animals [408]. For example, GM1 can partially protect against 6-OHDA treatment [409] and aging-related DA deficits [410] as well. However, studies on MPTP-treated non-human primates have shown that a short treatment with GM1 does not lead to any improvement [411], while a chronic treatment does have a positive effect [412], which might be restricted to the surviving DA neurons in the midbrain, rather than due to the prevention of cell death [413]. Mechanistically, GM1 treatment increases DA innervation, dopamine synthesis, and TH expression following an MPTP lesion [414,415,416,417,418,419,420,421,422,423]. Moreover, GM1 inhibits the inflammatory response triggered by 6-OHDA [424], protects against the toxic intracellular GPR37 aggregates observed in parkinsonism [425] and is involved in the internalization of α-synuclein into microglia [426]. Nonetheless, evidence for an α-synuclein-linked role of GM1 is controversial: in one study it was claimed that GM1 may accelerate α-synuclein aggregation [427] and the formation of proteinase-resistant α-synuclein [392], but other work demonstrated that it induces alpha-helical structure and inhibits or eliminates α-synuclein fibril formation (depending on the amount of GM1 present) [289,428]. It is also unclear whether membranes containing GM1 interact with α-synuclein [428,429]. Hence, GM1 is a promising candidate for PD treatment, but further clarification of its specific effects on α-synuclein is urgently needed.

Only a limited number of studies have analyzed the role of gangliosides other than GM1 in animal and cellular models. For instance, mice lacking GM2/GD2 synthase develop parkinsonism, which can be partially rescued by administration of GM1 [400,430]. However, GM2 accumulation, as seen in Tay Sachs and Sandhoff’s diseases, leads to α-synuclein aggregation [431]. Thus, both deficiency and excess of GM2 may lead to PD-like pathology. Likewise, GM3 accelerates α-synuclein aggregation [427] and regulates α-synuclein-induced channel formation in PC-containing membranes [301]. Furthermore, deletion of GD3 synthase, which decreases production of the pro-apoptotic GD3 ganglioside, protects against MPTP treatment in mice [432]. In contrast, ganglioside GT1b is neurotoxic in nigral DA neurons by triggering nitric oxide release from activated microglia [433]. The gangliosides GD3 and GT1b are unchanged and decreased in the SN of (male) PD patients, respectively [270]. Together, these results indicate that GM3, GD3, and GT1b play aggravating roles in PD pathology. Finally, 1-phenyl-2-decanoylamino-3-morpholino-1-propanol (PDMP, an inhibitor of glycosylceramide synthase that decreases ganglioside content) enhances α-synuclein toxicity, which can be rescued by ganglioside addition [434].

### 5.6. Sulfatides

Sulfatides, which are sulfated galactocerebrosides, form a group of lipids involved in protein trafficking, immune responses and neural plasticity, among others [435]. Higher levels of sulfatides have been detected in the plasma [385] and visual cortex [225] of PD patients, and in the SN of male PD patients [270]. Arylsulfatase A, an enzyme that breaks down sulfatides, has been linked to PD recurrence [436,437]. However, no changes or reductions in sulfatide levels have been described in lipid rafts from the frontal cortex of PD patients [33] and in brain samples from PD patients [438], respectively. Thus, most evidence points towards increased sulfatide levels in PD, although a number of studies have not confirmed this finding, suggesting patient, technique and/or tissue-type differences among the various investigations.

## 6. Sterols

**Figure 6:**
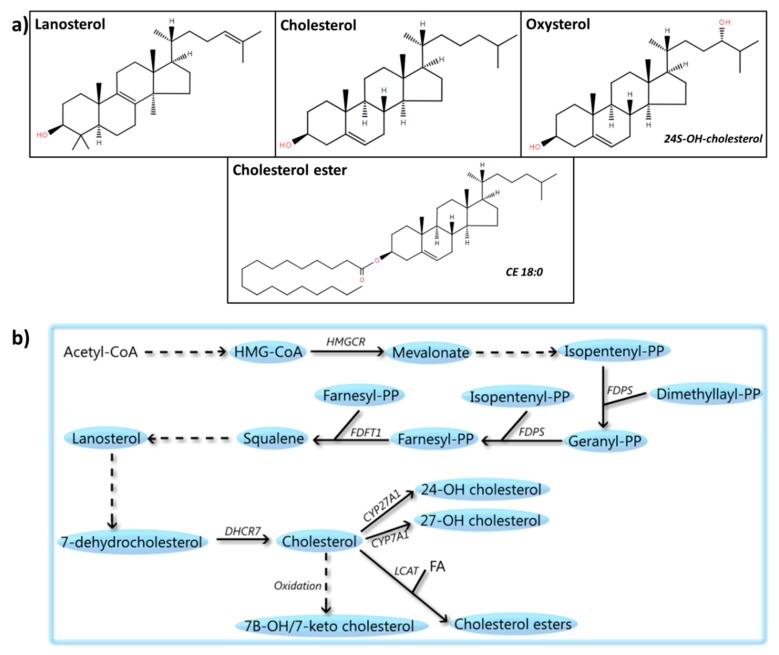
Sterols: structures and metabolic steps involved. (**a**) Schematic representation of the chemical structures of sterols, including lanosterol, cholesterol, oxysterols (24S-hydroxy-cholesterol), and cholesterol esters (CE 18:0). Chemical structures are adapted from the LIPID MAPS structure database [25]. (**b**) Schematic overview of steps involved in sterol metabolism. Acetyl-CoA is used to synthesize β-hydroxy β-methylglutaryl-CoA (HMG-CoA), which is converted into mevalonate by 3-hydroxy-3-methylglutaryl-coenzyme A reductase (HMGCR). Mevalonate is metabolized to isopentenyl-PP by a multistep process, followed by its conversion to geranyl-PP and farnesyl-PP by the enzyme farnesyl pyrophosphate synthase (FDPS). Two molecules of farnesyl-PP are condensed by squalene synthase (FDFT1) to create squalene, which is further metabolized to lanosterol and 7-dehydrocholesterol. Subsequently, cholesterol is synthesized from 7-hydrocholesterol by 7-dehydrocholesterol reductase (DHCR7). Finally, cholesterol can be oxidized to compounds such as 7-beta-hydroxycholesterol or 7-ketocholesterol. It can also be esterified to a fatty acid (FA) by phosphatidylcholine-sterol acyltransferase (LCAT) to create cholesterol esters or metabolized by the cytochrome p450 to produce compounds such as 24/27-hydroxycholesterol.

Sterols are amphipathic lipids synthesized from acetyl-CoA via the β-hydroxy β-methylglutaryl-CoA reductase pathway and containing a fused four-ring core structure (Figure 6). Sterols are known to play a role in immune cell function [439], influence membrane fluidity and permeability, and serve as signaling molecules and hormones [440], among others. Here we will review the current findings on sterols in PD, more specifically cholesterol, its precursors, CE, and oxysterols (Supplementary Materials Table S1).

### 6.1. Cholesterol

#### 6.1.1. Human Studies on Cholesterol

Cholesterol intake has been found to be negatively [29,441], positively [30,442], or not [31,443] correlated with PD risk. A meta-analysis indicates a lack of association between cholesterol intake and PD [444]. Lower plasma cholesterol has been associated with PD [229,232,445,446,447], and confirmed by a meta-analysis [238], and higher plasma cholesterol levels have been linked to reduced PD risk [235,448,449,450,451,452] and slower clinical progression of PD [453]. However, others, including a meta-analysis [454], have found no association between plasma cholesterol levels and PD [230,455] or PD risk [233,456]. Even higher plasma cholesterol levels in PD patients compared to controls [231,457] have been reported. The differential outcome of these studies could be attributed to factors such as age and gender, among others, since lower plasma cholesterol levels have been reported in PD male patients of more than 55 years compared to controls [458], a high total cholesterol baseline has been associated with increased risk of PD in subjects of 25–54 years (but not in those above 55) [459], and female PD patients seem to have higher cholesterol levels compared to male PD patients [460]. Thus, proper patient stratification is necessary to determine whether plasma cholesterol is associated with PD, which would point towards defects in cholesterol metabolism.

In PD patients, no significant changes in cholesterol levels have been observed in the putamen [383], SN [270] or frontal cortex lipid rafts [33], while elevated levels of cholesterol have been found in the visual cortex [225]. Finally, decreased cholesterol biosynthesis has been described in fibroblasts from PD patients [461]. The differences in these observations could be related to tissue or brain-region specificities, technique sensitivity, and/or choice of patients. Thus, validation studies and larger cohorts are needed to determine the relevance of cholesterol changes in PD patients and their pathology. Additionally, some studies [13,462,463] have found an association between PD and a SNP near the gene *SREBF1*, which encodes a transcription factor that regulates cholesterol biosynthesis, although other studies could not confirm the findings [464].

#### 6.1.2. Animal and Cellular Studies on Cholesterol

In animal and cell model studies, the link between PD and cholesterol has been demonstrated multiple times. For example, the cholesterol biosynthetic pathway controls *PRKN* expression [465], which in turn regulates fat (and cholesterol) uptake in *PRKN* mutant mice and human cells [466]. Additionally, *DJ-1* KO mouse embryonic fibroblasts and astrocytes display lower cellular (but not plasma [467]) cholesterol levels and impaired endocytosis [468], which can be rescued by increased membrane cholesterol [469]. In contrast, *GBA* KO and *PRKN* KO cells have increased cholesterol levels [470,471], and the N370S *GBA* mutation leads to cholesterol accumulation in lysosomes [472], while *LRRK2* KO rats have higher serum cholesterol levels [473]. Thus, cholesterol biosynthesis seems to be impaired in PD, but the direction of the change differs among PD etiologies, which could explain part of the variation observed in different studies with PD patients.

Increased cholesterol reduces cell death [474] and modulates presynaptic DA phenotype by increasing TH and VMAT2 expression in SH-SY5Y cells [475] and enhancing ligand binding of DAT and VMAT2 in the brains from rats and monkeys [476]. However, hypercholesterolemia seems to cause DA neuronal loss and oxidative stress in the SN and the striatum, leading to motor impairment [477,478,479]. Together with the observation that cholesterol treatment of (MPP+-treated) SH-SY5Y cells reduces their viability [480], this finding suggests that the effect of cholesterol levels on PD is dose dependent.

#### 6.1.3. Alpha-Synuclein and Cholesterol

Alpha-synuclein interacts with cholesterol [481] and cholesterol-containing vesicles [482], but it is unclear whether cholesterol facilitates the binding of α-synuclein to charge-neutral membranes [483,484]. Alpha-synuclein-cholesterol interaction seems to be associated with α-synuclein accumulation [474,485] and aggregation [486] and is a determining factor in α-synuclein’s ability to form pores [487,488]. Accordingly, reducing cholesterol levels leads to decreased α-synuclein accumulation and damage in the synapse [489,490,491]. Hence, high levels of cholesterol aggravate α-synuclein-associated pathology. Furthermore, α-synuclein potentiates cholesterol efflux [492], antagonizes cholesterol in lipid rafts [493], and enhances production of oxidative cholesterol metabolites [494]. Finally, A53T-α-synuclein-overexpressing mice have increased levels of serum cholesterol [239], while WT-α-synuclein-overexpressing mice have upregulation of genes involved in cholesterol biosynthesis in DA neurons from the SN [495], indicating a tight reciprocal relationship between α-synuclein and cholesterol metabolism.

#### 6.1.4. Statins

Statins are cholesterol-lowering drugs that have been described to decrease [496,497,498,499,500,501] or not affect [502,503,504] PD risk. Interestingly, lipophilic, but not hydrophilic, statins increase PD risk [505]. In the current discussion on the contradictory findings regarding the effects of statins not enough attention is paid to confounding factors such as statin indication, statin-type effects or immortal time bias (span of cohort follow-up during which the outcome under study cannot occur), and healthy user effects [506]. In animal and cellular models, atorvastatin pretreatment seems to prevent early effects of MPTP administration in rats [507], and lovastatin has neuroprotective effects against MPP+ and 6-OHDA [474,508] and ameliorates α-synuclein accumulation [509,510]. Similarly, simvastatin is neuroprotective against 6-OHDA and MPTP treatments [511,512,513,514] and increases dopamine content in the striatum [515]. However, negative effects of simvastatin and atorvastatin on MPP+-mediated toxicity have also been reported [516], which could be explained by the fact that statin lactones, one of the statin metabolites, are able to inhibit mitochondrial complex III [517], potentiating MPP+ toxicity.

### 6.2. Cholesterol Precursors

In PD patients, the cholesterol-synthesizing enzymes isopentenyl diphosphate isomerases 1 and 2 have been observed in LB from the SN of PD patients [518]. The natural cholesterol intermediate squalene seems to prevent toxicity in the striatum of 6-OHDA-treated mice [519], whereas α-synuclein accumulation enhances squalene production [242], which could be a cellular response to oxidative damage. A derivative of squalene, squalane, exacerbates 6-OHDA toxicity [519]. The naturally occurring cholesterol precursor lanosterol induces mitochondrial uncoupling and protects DA neurons from cell death in the nigrostriatal region of MPTP-treated mice [520]. Thus, cholesterol precursors seem to have a protective role in PD. Interestingly, inhibitors of both geranylgeranyl transferase (GGTI) and farnesyl transferase (FTI), enzymes that transfer the prenyl group geranylgeranyl or farnesyl to proteins, protect nigrostriatal neurons in MPTP-intoxicated mice [521].

### 6.3. CEs

The esters between cholesterol and fatty acids, CEs, are synthesized from excess cholesterol in the cytosol by the enzyme acetyl-coA acetyltransferase 1, a process that can be reversed by the enzyme cholesteryl ester hydrolase. In PD patients, reduced cholesterol esterifying activity has been detected in fibroblasts [461] and CE 20:5 is reduced in their visual cortex [222]. Interestingly, in *C. elegans* the ortholog of neutral cholesteryl ester hydrolase 1 attenuates α-synuclein neurotoxicity when sufficient CE is present, while knockdown leads to neurodegeneration [522]. However, GBA KO cells have increased levels of CE 15:1, 22:6, and 24:1 [470], which could reflect either a protective or a pathological mechanism.

### 6.4. Oxysterols

The products of cholesterol oxidation, 7beta- and 27-hydroxycholesterol, and 7-ketocholesterol, are elevated in plasma from PD patients [162]. Additionally, 27-hydroxycholesterol CSF levels are increased in a subgroup of PD patients [523] Moreover, increased cholesterol lipid hydroperoxides have been observed in the SN of PD patients [524]. The CSF levels of 24-hydroxycholesterol appear to be correlated with PD duration [523], but higher levels have also been observed in early stage PD [525]. Conversely, 24-hydroxycholesterol esters are reduced in plasma from PD patients [526]. Of note, TH levels are increased by 24-hydroxycholesterol [527], while 27-hydroxycholesterol seems to reduce TH expression and increases α-synuclein levels [527,528,529,530]. An unexpected finding was that both 24- and 27-hydroxycholesterol seem to protect against staurosporine-induced cell death [531]. Interestingly, oxysterols, and more specifically 24(S),25-epoxycholesterol, increase DA neuronal differentiation via liver X receptors in both mouse and human embryonic stem cells [532,533].

## 7. Lipoproteins

Lipoproteins transport triglycerides and cholesteryl esters. Together, these lipids form the core of the lipoprotein, which is further surrounded by glycerophospholipids and free cholesterol [534]. Lipoproteins are classified according to their density, and thus their composition, as high-density lipoproteins (HDL), intermediate-density lipoproteins (IDL), low-density lipoproteins (LDL) or VLDL. Here, we will specifically review the current findings concerning HDL, LDL and VLDL (Supplementary Table S1).

### 7.1. HDL

The assembly complex HDL is composed of proteins (around 40%, mainly apolipoprotein A1 (ApoA1), but also apolipoprotein C (ApoC), apolipoprotein E (ApoE), and apolipoprotein J (ApoJ)) and lipids (including around 30% of glycerophospholipids, 25% of cholesterol/CE, and 5% of TAG). The main biological role of HDL is in cargo transport, in particular of lipids and proteins, but it is now also known to bring miRNAs to recipient cells [535]. Lower plasma HDL and ApoA1 levels have been associated with earlier PD onset [536] and higher PD risk [237,537,538,539], and HDL levels are positively correlated with disease duration [540]. Plasma levels of HDL-cholesterol are lower [229,385,541] or not different [230,233,446,447,457] in PD patients compared to controls. This controversial relationship is complex, as both sex [460] and *APOE* polymorphisms [231] seem to affect HDL-cholesterol levels in PD patients.

### 7.2. LDL

About 20% of LDL consists of proteins (mainly apolipoprotein B (ApoB)) and the remainder consists of lipids (including about 22% of glycerophospholipids, 50% of cholesterol/CE, and 8% of TAG). High LDL-cholesterol levels in plasma are protective for PD and associated with preserved executive and fine motor functions in PD [452,455,457,542], while lower LDL-cholesterol levels are associated with higher PD risk [229,445,446,447,500,543,544]. One study reported that plasma LDL levels are not different between PD patients and controls [237]. A number of other studies have reported no difference in baseline LDL-cholesterol [230,497], and two meta-analyses have found no association [238,496]. Furthermore, in contrast to HDL, LDL-cholesterol levels do not differ between male and female PD patients [460,540]. However, one study reported increased LDL-cholesterol levels in PD patients compared to controls [231]. Interestingly, compared to controls PD patients seem to have higher levels of oxidized LDL [545], which is able to enter neuronal cells and elicit neurotoxicity [546]. Finally, male *DJ-1* KO mice have higher LDL-cholesterol levels in serum, which could be due to the fact that the LDLR is a transcriptional target of DJ-1 [467].

### 7.3. VLDL

Very low-density lipoproteins are mainly composed of lipids (including around 15% of glycerophospholipids, 20% of cholesterol/CE, and 60% of TAG) and only minor amounts of protein (around 5%, mainly ApoB and ApoC). Parkinson’s disease patients appear to have lower levels of both VLDL [230] and VLDL-cholesterol [231] than controls, but the role of VLDL in PD remains unclear.

## 8. The Cellular Lipidome

Above we have given an overview of the changes in lipid composition that have been observed in multiple studies involving PD patients, and animal and cellular PD models. The question arises what the significance of such changes is from a biological point of view. In mammalian cells, about 5% of the genes are involved in the generation and transport of an estimate of 10,000 individual lipid species [547,548], which have structural [549], signaling [549,550], and energy storage [17,18] roles. More specifically, above-mentioned molecules such as glycerophospholipids, sphingolipids, and sterols represent the main components of the cell’s plasma and mitochondrial membranes, endoplasmic reticulum, the Golgi complex, and endosomes. In a dynamic manner, lipid composition defines organelle identity [547], controls the recruitment of proteins, and lipid bilayer properties, such as thickness, elastic compression, and intrinsic curvature, can be an allosteric regulator of membrane protein function [551]. Alterations in membranes thus dynamically control important processes such as (synaptic) vesicle trafficking, endocytosis-exocytosis [552] or α-synuclein aggregation [553], processes that have already been associated with PD [554,555,556].

Lipids also play an important role in intracellular and intercellular signaling in the brain by direct interaction with receptors and other signal-transducing proteins [549,550] that regulate integral physiological processes linked to PD. For example, PUFA are involved in inflammation, neurogenesis, and neuroprotection [56]. Endocannabinoids are lipid-based retrograde neurotransmitters that modulate synaptic plasticity [557], and LPA modulates processes like proliferation, survival and migration [249]. Additionally, although most energy consumed by brain cells comes from glucose, lipids have been suggested to provide up to 20% of the total energy consumption of the adult brain [549,558,559]. Therefore, changes in lipid composition or content, such as the ones that have been described here for PD, can have vast consequences for key processes in the maintenance of normal neuronal and brain function. However, unlike what holds for genes and proteins, most lipid species cannot be associated with specific functions: their role is dictated by the concentration and location of individual lipid species, and, most importantly, by their interaction with other lipid species. Since most of the available information is a description of changes in lipid concentration, firm conclusions regarding the effects of these changes are hard to draw.

This lack of precise knowledge regarding the (patho)biological significance of lipidome abnormalities is predominantly caused by the fact that lipids form a vast and enormously complex group of biomolecules. This creates two major challenges. First, it is currently very difficult—if not impossible—to characterize all lipids present in the lipidome of a sample, due to limitations in the separation methods. This precludes simultaneous analysis of all lipid classes, which is especially hindered by the presence of isomeric (i.e., same mass) lipids. Second, no methodology is currently available to accurately determine the concentrations of the various lipid species [560]. This lack of information hampers the interpretation of lipidomic studies and the creation of reliable databases that, on its turn, impedes the identification of pathways in which a combination of lipid species plays a role [561].

As mentioned, not only their composition in the lipidome but also the tissue distribution and intracellular localization of individual lipids are crucial for their function, which makes it of great importance to develop techniques to identify and quantify lipids at the single-cell level and with the spatial organization of the cell still intact. These developments will help to elucidate the interplay of different lipid species in a time- and location-dependent manner both in health and disease. Indeed, it would allow us to obtain more information about (i) the lipidomes of various cell types, which are now identified in growing numbers within different tissues and organs by RNAseq [562], and (ii) the dynamic changes in lipidome composition that are associated with disease progression. Unfortunately, proper sample preparation, even more than the detection limits for lipids in mass-spectrometry, currently forms the biggest barrier to develop effective single-cell lipidomics [563]. Moreover, the interplay between lipids and other biomolecules necessitates the integration of lipidomics with other omics strategies [564].

It is also important to note that the lipidome composition is not only defined by the activity of genes involved in lipid metabolism, but also strongly depends on exogenous factors. These include (1) the direct dietary intake of lipids and lipid precursors from food, (2) life-style factors, i.e., exercise, sleep patterns, and intrinsic and extrinsic motivation factors that determine the choice of food composition, and (3) the effects of drugs that affect metabolism or cell behavior. For example, accumulating evidence suggests that tight bidirectional interactions exist between dietary lipids and composition and structure of the gastrointestinal tract microbiota [565,566,567]. This could be especially relevant for PD, since dysbiosis (i.e., the change in microbiota structure relative to that found in healthy individuals [568]), has been repeatedly observed in PD patients [569,570,571,572,573,574] from early stages of the disease onwards [575].

Filling in the current gaps in lipidomics technology and knowledge is crucial to exploit its potential to help us further understand the molecular mechanisms underlying PD, better define its stages and classification, and identify biomarkers, create dietary interventions, or perform compound screening, preclinical testing and monitoring of drug responses [576,577].

## 9. Conclusions

From this review, it is clear that a strong correlation exists between PD and abnormalities in lipid metabolism. More specifically, there is an association between PD and the levels of fatty acyls (SFA, MUFA, PUFA, a number of eicosanoids, and acylcarnitine), glycerolipids (MAG, DAG, and TAG), glycerophospholipids (PA, LPA, PE, PS, PC, LPC, PI, PIPx, PG, and CL), sphingolipids (sphingosine(-1P), ceramide, SM, cerebrosides, gangliosides, and sulfatides), sterols (cholesterol precursors, cholesterol, CE, and oxysterols) and lipoproteins (HDL, LDL, and VLDL). Furthermore, there is a conspicuous relationship between the folding, aggregation, and distribution of α-synuclein and the lipids that drive some of the neuropathological features of PD. Yet, it is presently unclear whether links exist between PD and some eicosanoids (eoxins, thromboxanes, oxoeicoanoids, hepoxilins, lipoxins, and epoxyeiconsatetraenoic acid), glycerophospholipids (lysoPE, lysoPS, lysoPI, lysoPG, lysoCL, and Bis(monoacylglycero)phosphate), sphingolipids (globosides), and lipoproteins (IDL).

One of the main concerns regarding the findings summarized in this review is that most lipid classes have not been consistently found to be associated with PD. Variables such as sex, age, PD etiology, specific DNA polymorphisms or the microbiome may have influenced the findings. Thus, proper stratification of PD patients is necessary to understand the biological implications of the lipid changes observed. Additionally, more accurate description of the lipid profiles of plasma, CSF and/or fibroblasts from PD patients will help to classify the patients more accurately.

A further concern is that most studies have focused on plasma levels of lipids, but these may not correlate with their brain levels, e.g., levels of ganglioside species are increased in plasma but not in brains of PD patients [270,385]. Thus, CSF (and brain) lipidomes of PD patients have to be determined to get insight into the actual pathological lipid composition and processes. Moreover, it is often unclear whether the changes in the levels of lipid species reflect a pathological or rather a compensatory mechanism. Finally, studies on cellular and animal PD models do not always show the same directionality of lipid level changes as found in studies on PD patients.

In conclusion, ample evidence for a central role of lipids in PD is available, but current data yield a picture that is still too fragmented. This hinders the unraveling of the specific pathological mechanisms in which lipids are involved. Technological advances to better characterize the lipidome and explore the functions of specific lipid species, together with additional studies on CSF and/or brain tissue from PD patients are now urgently needed to further our understanding of the pathobiology of the relationship between PD and lipids and will help us to identify biomarkers and druggable targets for the development of disease-modifying therapies for this devastating neurodegenerative disease.

## Supplementary Materials

The following are available online at http://www.mdpi.com/2073-4409/8/1/27/s1, Table S1: Lipid and lipoprotein levels in human PD body fluids and tissues, and their effects in animal/cellular models.
