# Supplementary material for: The Role of Lipids in Parkinson’s Disease
Source: Cells. 2019 Jan 7;8(1):27. doi: 10.3390/cells8010027 (PMC6356353; doi:10.3390/cells8010027)
Supplement: Supplementary file 1 [file cells-08-00027-s001.pdf]

Supplementary Table S1. Lipid and lipoprotein levels in human PD body fluids and tissues, and their effects in animal/cellular models. (=) No differences between PD patients and controls; (≠) Discordant between multiple studies; (↑) Increased in PD patients compared to controls; (↓) Decreased in PD patients compared to controls; (+) Positive effect on PD animal/cellular models; (-) Negative effect on PD animal/cellular models; (DA) Effect on dopaminergic neurons; [a] Lipid rafts studies; [b] Cytosolic fraction studies; CSF, Cerebrospinal fluid.

	Intake	Plasma	CSF	Brain								Animal/ cellular models
				Brain	Anterior cingulate cortex	Cerebellum	Frontal cortex	Primary visual cortex	Putamen	Substantia nigra	Temporal cortex	
Fatty acyls												
SFA	=						↑[a]				=	+ -
MUFA	≠										=	DA
PUFA					↑			=			=	+
Omega-3	↓											+
ALA	↓	↓										
EPA												+
DHA							↓[a]↑[b]					+
Omega-6	≠											
LA	≠	↓					↓[b]					↑ +
AA	≠	↓	↑				↓[a] =[b]					↑ +
Eicosanoids												
Prostaglandins			=							↑		↑ + -
Leukotriens												-
Epoxyeicosatrienoic acid												+
Isoprostanes		≠	=		↑					=		
Carnitine		↓										+
Acylcarnitine		≠	=									+
Glycerolipids												
MAG												+**
Endocannabinoids												
Anandamide			↑									+
2-AG												↑ +
CB1R									↑			≠
CB2R									↓	↑		≠

DAG		↓					↑	↑				
TAG		↓						↓				↓
Phospholipids												
PA		↑										
LysoPA												DA
PE		↓					↑[a]	↓		↓		
PS		↓					↑ =[a]	↑				↑ -
PC		↓										↓
LysoPC		↑										≠
PI							↑[a]					
PG							=[a]					
CL												≠
Sphingolipids												
Sphingosine												
S1P												+
Ceramide		≠			↓			↑				↑ + -
SM					↑↓	=		↑	=			
Cerebrosides		↑				=	↓[a]		=		=	
Gangliosides		↑										
GM1										↓		+
GM2						=			=			
GM3		↑				=			=			
NANA-3		↑										
GD3												
GT1b												
Sulfatides		↑		↓			↓[a]	↑				↑
Sterols												
Cholesterol	≠	≠					=[a]	↑	=			≠
Cholesterol precursors												+
Squalene												+
Squalane												-
Lanosterol												+
Cholesterol esters								↓				
Oxysterols										↑		
7beta-hydroxycholesterol		↑										

27-hydroxycholesterol		↑	↑									DA
7-ketocholesterol		↑										
24-hydroxycholesterol			↑									DA +
24(S),25-epoxycholesterol												
Lipoproteins												
HDL		↓										
HDL-C		≠										
LDL		=										
LDL-C		↓										
VLDL		↓										
VLDL-C		↓										
